# Supplementary material for: Effect of Training Load on Post-Exercise Cardiac Troponin T Elevations in Young Soccer Players
Source: Int J Environ Res Public Health. 2019 Dec 2;16(23):4853. doi: 10.3390/ijerph16234853 (PMC6926666; doi:10.3390/ijerph16234853)
Supplement: Supplementary file 1 [file ijerph-16-04853-s001.pdf]

International Journal of
*Environmental Research and Public Health*

MDPI

**Table 1.** Model coefficients.

| Variable | Parameter | Coefficient | MPE |
| --- | --- | --- | --- |
| **Time** | $\beta_0$ (Intercept) | 0.48 (0.26–0.72) | |
| $R^2$ = 64.4 (52.5–74.7) | $\beta_1$ = hs-cTnT$_{0h}$ | 0.17 (−0.11 to 0.44) | 84.4% |
| | $\beta_2$ = hs-cTnT$_{3h}$ | 1.4 (1.12–1.68) | 100% * |
| **Age (Years)** | $\beta_0$ (Intercept) | 0.48 (0.26–0.7) | |
| | $\beta_1$ = Post | 1.4 (1.11–1.68) | 100% * |
| $R^2$ = 68.7 (59.4–78.5) | $\beta_2$ = Age | 0.06 (−0.18 to 0.27) | 65.6% |
| | $\beta_3$ = Post × Age | 0.42 (0.13–0.74) | 98.7% * |
| **Tanner Stage** | $\beta_0$ (Intercept) | 0.48 (0.25–0.73) | |
| | $\beta_1$ = Post | 1.39 (1.08–1.74) | 100% * |
| $R^2$ = 63.8 (52.1–74.7) | $\beta_2$ = Tanner | 0.07 (−0.16 to 0.34) | 67.9% |
| | $\beta_3$ = Post × Tanner | 0.28 (−0.04 to 0.62) | 91.8% * |
| **Experience (Years)** | $\beta_0$ (Intercept) | 0.48 (0.27–0.68) | |
| | $\beta_1$ = Post | 1.4 (1.14–1.67) | 100% * |
| $R^2$ = 72.9 (64.3–81.5) | $\beta_2$ = Experience | 0.05 (−0.13 to 0.28) | 66.8% |
| | $\beta_3$ = Post × Experience | 0.5 (0.23–0.78) | 99.8% * |
| **BMI (kg/m$^2$)** | $\beta_0$ (Intercept) | 0.48 (0.22–0.73) | |
| | $\beta_1$ = Post | 1.39 (1.08–1.74) | 100% * |
| $R^2$ = 59.8 (46.2–72) | $\beta_2$ = BMI | 0.09 (−0.17 to 0.35) | 73% |
| | $\beta_3$ = Post × BMI | 0.03 (−0.3 to 0.37) | 56.2% |
| **HR Max (bpm)** | $\beta_0$ (Intercept) | 0.48 (0.26–0.7) | |
| | $\beta_1$ = Post | 1.4 (1.13–1.7) | 100% * |
| $R^2$ = 69.1 (59.3–79) | $\beta_2$ = HR max | −0.05 (−0.28 to 0.17) | 64.3% |
| | $\beta_3$ = Post × HR max | −0.43 (−0.71 to −0.11) | 99% * |
| **HR Aver (bpm)** | $\beta_0$ (Intercept) | 0.49 (0.21–0.78) | |
| | $\beta_1$ = Post | 1.34 (0.95–1.69) | 100% * |
| $R^2$ = 57.6 (42.2–71) | $\beta_2$ = HR aver | 0.07 (−0.2 to 0.36) | 65.6% |
| | $\beta_3$ = Post × HR aver | 0.09 (−0.31 to 0.45) | 66.4% |
| **rHR Aver (%)** | $\beta_0$ (Intercept) | 0.49 (0.23–0.76) | |
| | $\beta_1$ = Post | 1.34 (0.98–1.68) | 100% * |
| $R^2$ = 58.9 (44.4–72.3) | $\beta_2$ = rHR aver | 0.07 (−0.19 to 0.36) | 66.6% |
| | $\beta_3$ = Post × rHR aver | 0.17 (−0.2 to 0.52) | 78.4% |
| **HR Peak (bpm)** | $\beta_0$ (Intercept) | 0.49 (0.2–0.76) | |
| | $\beta_1$ = Post | 1.34 (0.99–1.71) | 100% * |
| $R^2$ = 56.6 (41.7–70.5) | $\beta_2$ = HR peak | 0.05 (−0.24 to 0.35) | 60.4% |
| | $\beta_3$ = Post × HR peak | −0.08 (−0.47 to 0.3) | 64.8% |
| **rHR Peak (%)** | $\beta_0$ (Intercept) | 0.49 (0.22–0.77) | |
| | $\beta_1$ = Post | 1.34 (0.98–1.7) | 100% * |
| $R^2$ = 57 (42–71.6) | $\beta_2$ = rHR peak | 0.06 (−0.22 to 0.37) | 63% |
| | $\beta_3$ = Post × rHR peak | 0.03 (−0.38 to 0.4) | 54% |
| **Time HR-Z5 (min)** | $\beta_0$ (Intercept) | 0.5 (0.23–0.78) | |
| | $\beta_1$ = Post | 1.3 (0.97–1.68) | 100% * |
| $R^2$ = 60.7 (45.6–73.7) | $\beta_2$ = Time HR-Z5 | 0.04 (−0.24 to 0.33) | 60% |
| | $\beta_3$ = Post × Time HR-Z5 | 0.31 (−0.04 to 0.67) | 92.5% * |
| **RPE (UA)** | $\beta_0$ (Intercept) | 0.48 (0.27–0.72) | |
| | $\beta_1$ = Post | 1.39 (1.1–1.72) | 100% * |
| $R^2$ = 66 (53.8–76) | $\beta_2$ = RPE | 0.07 (−0.17 to 0.3) | 67.1% |
| | $\beta_3$ = Post × RPE | 0.34 (0.02–0.66) | 96.8% * |
| **Dist Abs (m)** | $\beta_0$ (Intercept) | 0.49 (0.24–0.72) | |
| $R^2$ = 70.1 (58.8–80.8) | $\beta_1$ = Post | 1.34 (1.01–1.63) | 100% * |
| | $\beta_2$ = Dist abs | 0.01 (−0.24 to 0.24) | 53% |

| | | | |
|---|---|---|---|
| | $\beta_3$ = Post × Dist abs | 0.52 (0.22–0.85) | 99.4% * |
| **Dist Rel (m/min)** | $\beta_0$ (Intercept) | 0.49 (0.26–0.72) | |
| | $\beta_1$ = Post | 1.34 (1.06–1.67) | 100% * |
| $R^2$ = 69.8 (58.7–80.1) | $\beta_2$ = Dist rel | 0.01 (−0.23 to 0.24) | 54% |
| | $\beta_3$ = Post × Dist rel | 0.52 (0.21–0.83) | 99.6% * |
| **Speed Aver (km/h)** | $\beta_0$ (Intercept) | 0.49 (0.24–0.71) | |
| | $\beta_1$ = Post | 1.34 (1.05–1.63) | 100% * |
| $R^2$ = 71.7 (61.3–81.5) | $\beta_2$ = Speed aver | 0.02 (−0.23 to 0.25) | 54.6% |
| | $\beta_3$ = Post × Speed aver | 0.55 (0.24–0.85) | 99.8% * |
| **Speed Peak (km/h)** | $\beta_0$ (Intercept) | 0.5 (0.22–0.77) | |
| | $\beta_1$ = Post | 1.34 (0.98–1.66) | 100% * |
| $R^2$ = 60 (45.6–73.7) | $\beta_2$ = Speed peak | −0.05 (−0.32 to 0.24) | 60.3% |
| | $\beta_3$ = Post × Speed peak | 0.29 (−0.06 to 0.66) | 90.3% * |
| **Time Speed-Z4 (min)** | $\beta_0$ (Intercept) | 0.49 (0.22–0.77) | |
| | $\beta_1$ = Post | 1.34 (1–1.68) | 100% * |
| $R^2$ = 60.2 (45–73.1) | $\beta_2$ = Time SP-Z4 | −0.04 (−0.32 to 0.23) | 59.6% |
| | $\beta_3$ = Post × Time SP-Z4 | 0.29 (−0.07 to 0.64) | 90.4% * |
| **Dist Speed-Z4 (m)** | $\beta_0$ (Intercept) | 0.49 (0.21–0.74) | |
| | $\beta_1$ = Post | 1.34 (1.02–1.71) | 100% * |
| $R^2$ = 60.4 (46.6–74.3) | $\beta_2$ = Dist SP-Z4 | −0.04 (−0.32 to 0.24) | 59% |
| | $\beta_3$ = Post × Dist SP-Z4 | 0.3 (−0.06 to 0.64) | 90.5% * |
| **Edwards' TL (UA)** | $\beta_0$ (Intercept) | 0.49 (0.22–0.78) | |
| | $\beta_1$ = Post | 1.34 (1–1.71) | 100% * |
| $R^2$ = 58.1 (42.9–71) | $\beta_2$ = TL | 0.06 (−0.21 to 0.35) | 63.7% |
| | $\beta_3$ = Post × TL | 0.13 (−0.25 to 0.49) | 72% |
| **Session RPE (UA)** | $\beta_0$ (Intercept) | 0.48 (0.25–0.71) | |
| | $\beta_1$ = Post | 1.39 (1.09–1.7) | 100% * |
| $R^2$ = 65.8 (54.8–76.7) | $\beta_2$ = s-RPE | 0.07 (−0.18 to 0.31) | 68.2% |
| | $\beta_3$ = Post × s-RPE | 0.34 (0.02–0.67) | 96.6% * |
